# How to enhance the well-being of healthcare service providers and their patients? A mindfulness proposal

**DOI:** 10.3389/fpsyg.2015.00276

**Published:** 2015-03-17

**Authors:** M. Joseph Sirgy, Pamela A. Jackson

**Affiliations:** ^1^Department of Marketing, Pamplin College of Business, Virginia Polytechnic Institute and State University (Virginia Tech)Blacksburg, VA, USA; ^2^Department of Psychology, Radford UniversityRadford, VA, USA

**Keywords:** mindfulness, healthcare service providers, patients, clinical situations, clinician-patient interface

The notion of *mindfulness* is key to developing ideas that can address how healthcare service providers (e.g., clinicians) can effectively enhance their own well-being in the workplace, and by doing so, increase the well-being of their patients. The seminal definition of mindfulness is “paying attention in a particular way: on purpose, in the present moment, and nonjudgmentally” (Kabat-Zinn, 1994, p. 4). Linehan (1993) argued that mindfulness involves six skills: three skills involve *what* the person is doing (observing, describing, and participating) and three skills relate to *how* the person is doing (non-judgmentally, focused attention, and performance quality). Another way to illustrate mindfulness is to make reference to its opposite: lack of awareness about current experience and a preoccupation with the past (rumination) or the future (worry). Therefore, mindfulness is both a skill and a way of being that can be cultivated through mindful meditation practices—formal practices such as sitting meditation or yoga, and informal practices such as eating, walking, and driving meditation.

Research has found that meditation practice of any sort may result in increased mindfulness (Tanner et al., 2009). Medical science has amassed much evidence that demonstrates the salutary effects of mindfulness on physical health (e.g., Keyworth et al., 2014). Also, recent reviews of the literature support the benefits of mindfulness practices to well-being (e.g., Brown et al., 2007; Galante et al., 2014).

We propose that a formal program be developed to provide clinicians and other healthcare service providers with structured training in mindfulness/meditation techniques, possibly during their initial education, but definitely during continuing education opportunities. For decades, theorists have proposed that meditation practice may have a positive impact on the healthcare service provider resulting in a stronger working alliance with patients (e.g., Shapiro and Carlson, 2009). Given the evidence of mindfulness-based interventions for patients (e.g., Seagal et al., 2002), researchers have begun to explore the impact of similar interventions on healthcare service providers (e.g., Greason and Cashwell, 2009). Based on a review of the research literature on counselor wellness, Cashwell et al. (2007) suggested that mindfulness practice may be beneficial for the therapist. The benefits included enhanced attention and concentration, expanded affect tolerance and acceptance, greater self-awareness and empathy, greater effectiveness in counseling, and greater therapist well-being. Bond et al. (2013) found that an 11-week elective course in yoga and meditation significantly improved scores on self-regulation and self-compassion scales in first and second-year medical students (pre-measures were administered 1 week prior to the commencement of the course and post-measures were administered 1 week after the conclusion of the course). Goodman and Schorling (2012) reported significant decreases in work-related burnout as well as increases in self-perceived mental and physical well-being following mindfulness-based interventions. Their study involved 93 healthcare service providers, including physicians, nurses, psychologists, and social workers. Shapiro et al. (2007) found that mindfulness training among mental health professionals decreased anxiety and increased self-compassion. Greason and Cashwell (2009) found that meditation practiced by student counselors produced greater empathy and ability to control attention in therapy sessions. Schure et al. (2008) reported similar findings related to counselors in training. The measures involved physical, emotional, mental, spiritual, and interpersonal well-being; these measures were administered in the context of a 15-week/3-credit academic course on stress reduction. Again, the Greason and Welfare (2013) reported that counselor mindfulness helped cultivate empathy and improved the working alliance with their clients. This study involved both counselors and their clients and measures were administered to both parties in each dyad. All of the studies cited above measured the effects of meditation/mindfulness practices on well-being almost immediately following the training. This is a drawback that should be addressed, hence the current proposal of a program that would include follow-up measures to assess not only well-being but the continued use of the mindfulness techniques.

We suggest here that once a healthcare service provider participates in the initial training to learn how to meditate and commits to practicing the techniques, further training might emphasize how to incorporate mindfulness into the clinical setting on an ongoing basis. Goldberg et al. (2014) found that the amount of time spent practicing mindfulness techniques was not important to later outcomes, but the quality of practice did have a significant impact. Therefore, once the healthcare service provider has mastered the technique, they may be able to practice a short, directed mantra during, or in between, stressful clinical situations, and this may be sufficient to insulate the service provider, as well as foster better quality of care for the patient.

Given the varied forms of meditations to achieve mindfulness and a higher state of well-being, the question arises: What form of meditation might be most effective in enhancing the well-being of the service provider and under what conditions? What follow are some hypotheses that can be tested in future research.

The manner in which healthcare service providers interact with their patients is likely to influence the well-being of both the service provider and their patients. These situations can be viewed along a temporal dimension capturing various points in time of the interface between the clinician and the patient. These situations include patient diagnostics/prognostics, patient treatment and progression, and two possible outcome situations: one involving the patient recovering and the other resulting in a more negative outcome. We hypothesize that the well-being effectiveness (for both the service provider and the patient) of a given meditation technique is dependent on the situation within the clinician-patient interface. Specifically,

- Situations involving patient *diagnostics/prognostics* may require meditation to relinquish negative thoughts and insight meditation. A meditation to *relinquish unwholesome thoughts*: disempowering negative thoughts and their corrosive effects on the mind and body by replacing the negative thoughts with positive ones and reflecting on the results of the negative thoughts (e.g., Epstein, 2004). Typically, in a diagnostics/prognostics situation, the patient is likely to feel anxious about the uncertainty of their situation. The patient may anticipate the worst possible scenario. This anxiety and negative thinking is likely to spill over to the clinician generating ill-being. Meditation designed to relinquish unwholesome thoughts can be very helpful in this stage. Ensuring that the clinician remains positive should not only help the clinician but also may reduce possible negativity in the patient in this situation.
- Situations involving *disease or disorder progression* may also call for *compassion and kindness* meditation techniques. During the treatment phase, the patient is likely to experience anger, anxiety, depression, and a host of other negative emotions. These negative emotions experienced by the patient are likely to spill over to the service provider and create ill-being. As such, what should help both the patient and the service provider is an authentic demonstration of compassion and kindness toward the patient; hence, compassion and kindness meditation (as practiced by the service provider) may be effective here too. Compassion meditation is the mindful practice of facing suffering, tragedy, and pain of others with empathy and without aversion or attachment (e.g., Armstrong, 2011). A systematic review and meta-analysis of the research (Galante et al., 2014) demonstrated that kindness-based meditation provides significant benefits for the health of individuals through its effects on well-being and social interaction. Research on loving-kindness meditation has shown that it increased other-focused concern, as well as self-compassion, in non-clinical samples, which suggests that it may do so in the healthcare service provider as well (Boellinghaus et al., 2012).
- Outcome situations may be quite positive or very negative. Situations involving patients *overcoming the disease or arresting the disorder progression* may call for *gratitude meditation*. In such situations, both patient and service provider may feel much emotional relief. This emotional relief can be used to further enhance the well-being of both the patient and the service provider by practicing gratitude meditation. This type of meditation involves the mindful practice of expressing good fortune and acknowledging the people and circumstances that led to this good fortune (e.g., Chan, 2010). Having the clinician practice gratitude meditation should not only enhance the well-being of the service provider but the patient too. The service provider, after practicing this type of meditation, is likely to manifest his or her feelings of gratitude in the presence of the patient, who in turn is likely to embrace these feelings as well.
- Situations involving patients *succumbing to the disease* or *dealing with a life-long disorder* may call for meditation techniques such as *insight* and *pain*. In such situations the patient is likely to experience fear and the inevitability of premature death or long-term disability (e.g., Gross, 2013). To deal with these negative emotions, the service provider should practice insight meditation. Insight meditation serves to clear the mind by accepting the notion of transience, impermanence, and imperfection (e.g., Goldstein, 1987). This mind set could also influence the patient. Here the service provider, after practicing this type of meditation, is likely to communicate with the patient about spiritual notions of transience, impermanence, and imperfection, which may help the patient feel more accepting of their condition. Heightened awareness and acceptance of existing experience should serve to interrupt the maladaptive, automatic response reflective of negative emotions (e.g., Seagal et al., 2002). Furthermore, the patient also is likely to experience much pain. Hence, pain meditation to help the patient may be effective for both the service provider and the patient. Pain meditation involves the mindful practice of releasing anger toward pain and accepting the situation in moments of pain (e.g., Kabat-Zinn, 1994). The service provider could suggest pain meditation to the patient, and possibly practice the technique together to help the patient with his or her pain and suffering.

As mentioned, these are essentially hypotheses that can be tested using experimental or longitudinal studies. We hope that the proposed ideas pave the way to a research program designed to identify situational, personality, and cultural moderators that can help us explain and develop the well-being effectiveness of various meditation techniques to increase mindfulness for clinicians and other health service providers. These ideas are offered in that spirit.

## Conflict of interest statement

The authors declare that the research was conducted in the absence of any commercial or financial relationships that could be construed as a potential conflict of interest.
